# Food insecurity and employment income: considerations regarding gender inequalities

**DOI:** 10.1017/S1368980025101201

**Published:** 2025-09-25

**Authors:** Aléxia Vieira de Abreu Rodrigues, Eloah Costa de Sant’Anna Ribeiro, Rosana Salles-Costa

**Affiliations:** 1 Postgraduate Program in Nutrition, Josue de Castro Nutrition Institute, Federal University of Rio de Janeiro, Rio de Janeiro, Brazil; 2 Department of Social Nutrition, Josue de Castro Nutrition Institute, Federal University of Rio de Janeiro, Rio de Janeiro, Brazil

**Keywords:** Employment, Gender equity, Family food insecurity

## Abstract

**Objective:**

To investigate the associations among income from work, the gender of the reference person, family and food insecurity (FI).

**Design:**

This quantitative study used nationally representative data from the 2018 Brazilian Family Budget Survey.

**Setting:**

The analyses estimated levels of food security and insecurity measured by the Brazilian Food Insecurity Scale according to labour income determined by the minimum wage per capita and the sex of the reference person (female/male). The logistic regression model measured the interaction between work income and gender in association with household FI.

**Participants:**

Brazilian families living in permanent households with at least one resident earning income from employment (*n* 48 625).

**Results:**

Households headed by women and with labour income ≤ ¼ minimum wage per capita had the highest percentage of moderate/severe FI (29·7 %). In these families and households with lower levels of employment income headed by men, the highest probabilities of moderate/severe FI were observed, at 10·8 and 9·6, respectively, compared with families with higher levels of employment income headed by men.

**Conclusions:**

Lower employment income contributes to FI in families, especially those that are headed by women. The socialisation of care work and the reduction in paid labour hours contribute to greater access to the labour market for women and a lower likelihood of FI.

The FAO of the UN has estimated that in the year 2022, food insecurity (FI) affected 2·4 billion people worldwide, with the greatest effect on women^(1)^. Approximately 28 % of women have moderate/severe FI compared with 25·4 % of men^(1)^. Furthermore, the latest FAO report indicated the presence of a persistent gender gap, especially in the 3 years prior to the report (2017–2019) and following the COVID-19 outbreak (2020)^(1)^.

In Brazil, the 2018 national survey, which was conducted before the pandemic, revealed an alarming increase in the proportion of families with severe FI^(2–4)^, especially those headed by women^(5–7)^. With the outbreak of the COVID-19 pandemic and the worsening of social inequalities, 19·3 % of families with a female head may have gone hungry due to severe FI, whereas 11·9 % of families with male heads may have experienced severe FI^(8)^. A new investigation focused on the last quarter of 2023 indicated that despite the decline of FI in Brazil, families headed by women experienced the most severe conditions of FI in a greater proportion (10·8 %) than families headed by men (7·8 %), which indicates gender inequality in access to food^(9)^.

Among Latin American countries, Brazil was a pioneer in the use of an experience-based measure, namely, the Brazilian Food Insecurity Scale (in Portuguese: Escala Brasileira de Insegurança Alimentar – EBIA), for the national monitoring of FI^(10)^. Although a significant reduction in FI was recorded in Brazil between 2004 and 2013, in 2018, severe FI increased by 40 % in households with a female reference person^(4)^. The increase in FI related to the global economic crisis that has affected Brazil since 2015 resulted in fiscal austerity measures that have discontinued social, food and nutritional security policies, thereby compromising access to food for the populations most vulnerable to FI^(11)^. In the same period, Brazil had the second highest prevalence of perceived gender inequality (77 %) among Latin American countries, inferior only to the gender inequality perceived by Colombian families (80 %)^(7)^.

Women are more affected in crisis contexts when there is a high increase in unemployment, less access to income and greater vulnerability to FI^(5–8)^. A lack of income to buy adequate food in sufficient quantities is the main factor in FI^(12–14)^. Particularly for women, the increased burden of caring for dependents can contribute to limited access to employment income and to FI^(8)^. Nationally representative data from the National Continuous Household Sample Survey (*Pesquisa Nacional por Amostras de Domicílios Contínua* – PNADc) reveal that female heads of single-parent families have significantly lower per capita employment income gains^(15)^ and are less available for full-time employment^(16)^.

Women perform the majority of unpaid reproductive labour, and their insertion and retention in paid labour are influenced by poverty^(16)^. This article is based on the concept of the sexual division of labour resulting from social power relations between genders^(17,18)^. Studies that debate the sexual division of labour can contribute to understanding the factors that contribute to gender inequalities and FI by prioritising productive labour for men and reproductive labour for women^(17)^.

This study is the first to use data from the 2018 Family Budget Survey (*Pesquisa de Orçamentos Familiares* – POF), which is a baseline survey representative of the Brazilian population, to assess FI and household income from residents’ employment. This study aims to evaluate employment income in relation to the sex of the family’s reference person.

## Methods

### Design

A cross-sectional quantitative study was conducted to analyse data from the 2018 Family Budget Survey (POF), which was conducted by the Brazilian Institute of Geography and Statistics (*Instituto Brasileiro de Geografia e Estatística –* IBGE). The POF is a nationally representative survey and constitutes the broadest investigation of the income, expenses and consumption of Brazilian families^(19)^. The current research uses the master sample, which was developed by the IBGE for the Household Survey System^(19,20)^. The POF sample was selected through two stages of cluster sampling (geographical and statistical stratification), and simple random sampling was used to select households^(19)^.

### Sample

The POF data collected between June 2017 and July 2018 via a trained team included 57 920 households that were interviewed throughout the national territory. The sample used for the current study comprised 83·9 % of households (*n* 48 625), that is, those that presented information on employment income.

### Food insecurity

Brazilian-scale FI (*Escala Brasileira de Insegurança* Alimentar – EBIA) was used to classify households according to food security (FS) and three levels of FI (mild FI, moderate FI and severe FI). The EBIA that has been validated for use within the Brazilian population consists of fourteen items, and each affirmative answer adds one point to the FI classification of households. In households with only residents over 18 years of age (adults and seniors), eight questions were asked; the remaining items referred to the presence of children and adolescents^(10)^. Cut-off points were established for households with or without children and adolescents under 18 years of age^(10)^. In the absence of affirmative answers, a household was classified as FS^(10)^. In this study, EBIA classification was modelled into three categories that represented the most serious levels (FS, mild FI, moderate/severe FI).

### Employment income

The POF evaluated employment income as the value of each resident of a household who answered the questionnaire POF 5 (job and individual income). The analysis of employment income considered salary information from the respondent’s main job (i.e. the resident’s only job, the one to which he or she dedicated the most hours per week or the job with the highest income). The mean employment income of all working residents of the household was calculated and categorised into multiples of the minimum wage per capita (MWPC), ≤ ¼ MWPC, > 1/4 ≤ 1/2 MWPC, > 1/2 ≤ 1 MWPC and > 1 MWPC. In January 2018, which served as the POF reference date, the value of the minimum wage in Brazil corresponded to R$954·00 (Brazilian real), which was equivalent to US$298·12 (US dollars) according to the exchange rate for the same period^(19)^.

### Gender

The gender of the reference person was investigated via the biological sex criterion of ‘female or male’^(19)^.

### Covariates

This study considered information such as the location (region (north, northeast, southeast, south, Midwest) and area (urban, rural) of households), the presence of residents < 10 years old in the household (yes; no) and the receipt of the cash transfer programme (Bolsa Família Program/BFP) (yes; no). In addition, the following information about the reference person or head of household was taken into account: self-reported race/skin colour (white, black, brown), schooling (< 8 years; ≥ 8 years) and marital status^(19,21)^.

In the IBGE surveys, the reference person, or the head of the household, was considered by the interviewers to be the individual who contributed most to the family income. In this sense, the interviewee in the survey was usually the reference person in the household, but when he or she was absent, the oldest person in the household was interviewed^(19,21)^.

### Statistical analysis

The analyses were conducted in STATA version 16·0. Considering the complex sampling design and the expansion factors provided by IBGE, we adopted the Survey Data Analysis command (prefix *svy*). The prevalence and corresponding 95 % CI of different levels of FS and FI were estimated.

In this study, EBIA classification was modelled as a three-level variable (FS, mild FI and moderate/severe FI). Statistical significance was considered based on the analysis of non-overlapping CI values (95 % CI) to identify the correlations among FS and FI levels and sociodemographic covariates considered^(22,23)^.

**Fig. 1:**
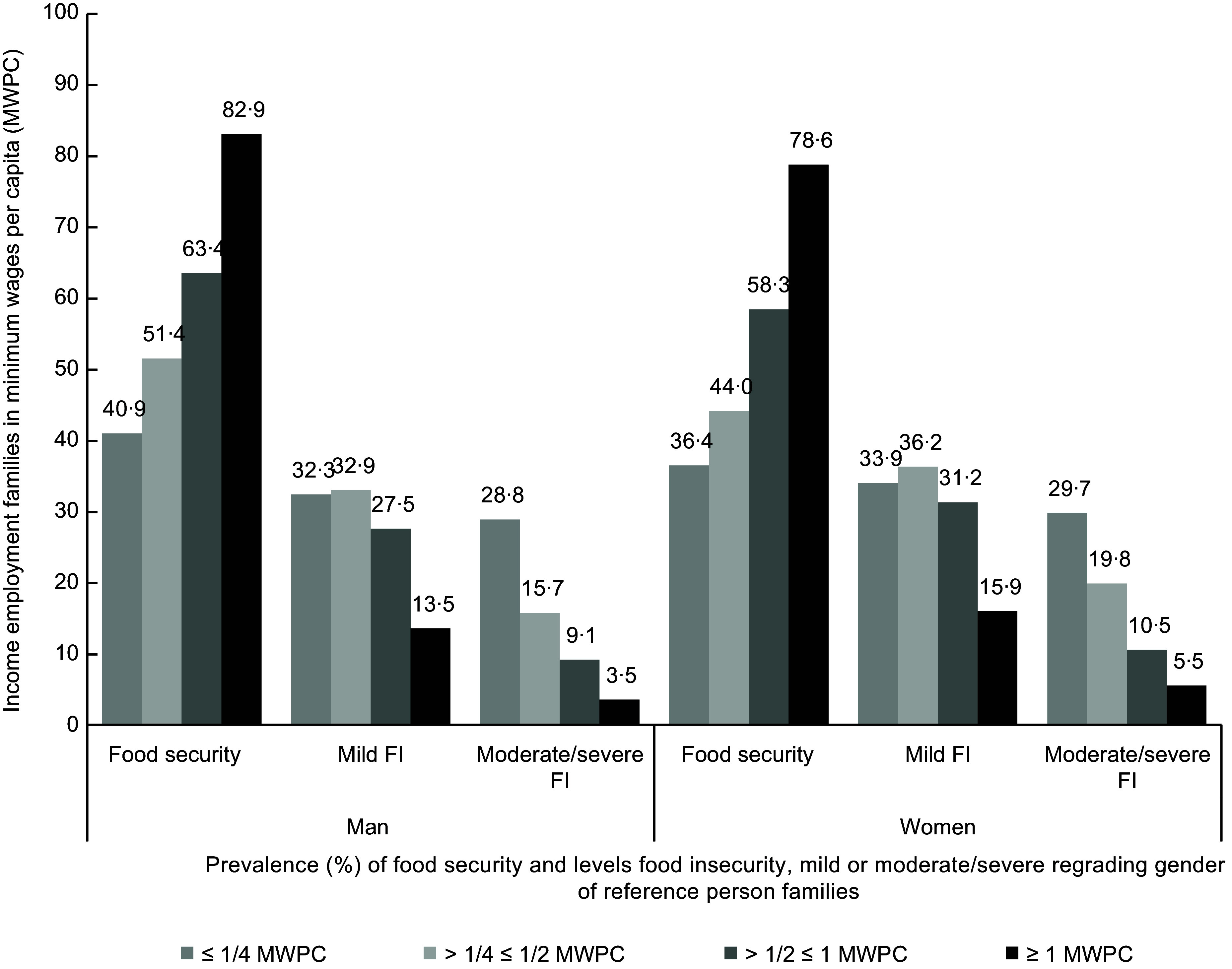
Prevalence of food security and levels of food insecurity (FI) regarding the income of employment, minimum wages per capita (MWPC) and gender of the reference person. Family Budget Survey – 2018.

The MWPC categories and gender of the reference individuals (Fig. 1) were compared in terms of FS and FI levels. Logistic regression models that considered the interaction between employment income and the gender of the family reference person were used to explore the association with FI. The models were adjusted by the covariates that were significantly associated with the levels of FS and FI in the descriptive analysis (Table 1). The interaction of the highest employment income (> 1 MWPC) and a male reference person was adopted as a reference category; that is, the other employment income profiles and the gender of the reference person were evaluated according to the previous status, including other categories of employment income in male-headed households. The model was adjusted by region, area, colour/skin colour, marital status, education, children < 10 years old and cash transfer BFP. After the final model was adjusted, the probabilities of the FI results were predicted according to employment income and the gender of the reference person in the household.

**Table 1.** Sociodemographic characteristics of Brazilian families, with at least one resident with income from work, according to food security (FS) and levels of food insecurity (FI), mild or moderate/severe. Family Budget Survey – 2018

	FS		Mild FI		Moderate/severe FI	
Variables	%	95 % CI	%	95 % CI	%	95 % CI
Region*						
South	79·4	77·9, 80·7	15·2	14·2, 16·4	5·4	4·7, 6·2
Nordeste	49·7	48·5, 50·8	29·8	28·9, 30·7	20·5	19·6, 21·5
Sudeste	68·8	67·4, 70·2	22·5	21·5, 23·6	8·6	8·0, 9·4
North	43·0	41·0, 45·1	31·8	30·0, 33·6	25·2	23·5, 26·9
Mild-west	64·8	62·6, 67·0	23·2	21·5, 25·1	12·0	10·7, 13·3
Area*						
Urban	64·8	64·0, 65·7	23·5	22·9, 24·2	11·6	11·2, 12·1
Rural	53·6	51·9, 55·2	27·2	26·0, 28·3	19·3	18·0, 20·6
Income of employment in minimum wages per capita (MWPC)						
≤ ¼ MWPC	38·7	37·3, 40·1	33·1	31·9, 34·3	28·2	27·0, 29·5
> ¼ ≤ ½ MWPC	48·4	46·8, 50·0	34·2	32·8, 35·7	17·4	16·2, 18·6
> ½ ≤ 1 MWPC	61·4	60·1, 62·7	28·9	27·7, 30·1	9·7	9·0, 10·4
> 1 MWPC	81·5	80·5, 82·4	14·3	13·5, 15·2	4·2	3·8, 4·6
Receipt of Bolsa Família Program*						
Yes	31·9	30·4, 33·4	37·0	35·7, 38·3	31·1	29·7, 32·5
No	68·4	67·6, 69·2	21·9	21·3, 22·5	9·7	9·3, 10·1
Presence of residents < 10 years old in the households*						
Yes	52·9	51·7, 54·0	32·7	31·7, 33·7	14·4	13·7, 15·2
No	67·9	67·1, 68·8	20·1	19·5, 20·8	11·9	11·4, 12·4
Profile of the family reference person						
Gender						
Women	58·4	57·4, 59·5	26·2	25·3, 27·1	15·3	14·7, 16·0
Men	66·8	65·9, 67·7	22·4	21·7, 23·2	10·8	10·3, 11·3
Marital status*						
With spouse	64·1	63·2, 65·0*	24·8	24·1, 25·5*	11·1	10·6, 11·6*
Without spouse	61·9	60·8, 63·0*	22·7	21·8, 23·5*	15·4	14·7, 16·1*
Education level*						
≤ 8 years	55·4	54·5, 56·4	26·5	25·7, 27·2	18·1	17·4, 18·8
> 8 years	69·3	68·3, 70·2	22·1	21·4, 22·9	8·5	8·1, 9·0
Race/skin colour*						
White	73·9	73·0, 74·9	18·6	17·8, 19·4	7·4	7·0, 7·9
Brown	54·1	52·2, 55·9	28·9	27·3, 30·6	17·0	15·7, 18·4
Black	54·6	53·6, 55·6	28·5	27·6, 29·3	16·9	16·3, 17·6

Minimum wage: USD 298·12 – US dollar (BRL 954·00 – Brazilian real). *Statistical significance was considered based on the analysis of non-overlapping CI values (95 % CI).

## Results

Rural families in the northern region were more vulnerable to FI at any level of severity. In relation to other sociodemographic characteristics, families that received BFP had a lower per capita family income stratum (MWPC) and had at least one child < 10 years old, a woman or a person with ≤ 8 years of education, and families of black race/skin colour were more vulnerable to FI at all levels. Furthermore, families headed by people without a spouse were found to have a higher prevalence of moderate/severe FI (15·4 %–95 % CI: 14·7, 16·0) (Table 1).

Considering the interaction between work income, the gender of the reference person and FI (Fig. 1), families with women as the reference person were more vulnerable to mild FI and moderate/severe FI regardless of the MWPC level (*P* value < 0·001). Although FS was related to the highest level of MWPC in both gender categories, the prevalence was higher among male-headed households. With respect to severe levels of FI, the lower the per capita income expressed in the MWPC, the greater the prevalence of FI among female-headed households (29·7 %) compared with male-headed households (26·8 %).

The model adjusted for the potential confounding factors for FI (Table 1) indicated that the lower the employment MWPC value, the higher the OR value in families headed by women (*P* value < 0·001). For the group with the lowest MWPC (≤ ¼ MWPC), the OR value was 9·36 times greater for families with moderate/severe FI than for families with high MWPC values (Table 2).

**Table 2.** OR income of employment and gender of reference person between levels of food insecurity (FI), mild or moderate/severe. Family Budget Survey – 2018

	Mild FI		Moderate/severe FI	
Income of employment (MWPC) and gender of reference person	OR adjusted	95 % CI	OR adjusted	95 % CI
≤ 1/4 MWPC and women	3·74	3·27, 4·30	9·36	7·81, 11·20
≤ 1/4 MWPC and men	3·05	2·67, 3·47	6·95	5·82, 8·29
> 1/4 ≤ 1/2 MWPC and women	3·78	3·30, 4·34	6·54	5·37, 7·97
> 1/4 ≤ 1/2 MWPC and men	2·80	2·46, 3·18	4·22	3·51, 5·08
> 1/2 ≤ 1 MWPC and women	2·83	2·48, 3·23	3·30	2·72, 3·97
> 1/2 ≤ 1 MWPC and men	2·20	1·95, 2·48	2·51	2·10, 3·00
> 1 MWPC and women	1·30	1·14, 1·50	1·66	1·35, 2·04
> 1 MWPC and men*	1·00		1·00	

MWPC, minimum wage per capita.

Adjusted for region, area, receipt of Bolsa Família Program, presence of residents < 10 years old in the households, marital status, education level and race/skin colour based on statistical significance in relation to the analysis of non-overlapping CI values (95 % CI) on Table 1.

Minimum wage: USD 298·12 – US dollar (BRL 954·00 – Brazilian real).

*Reference category.

**Fig. 2:**
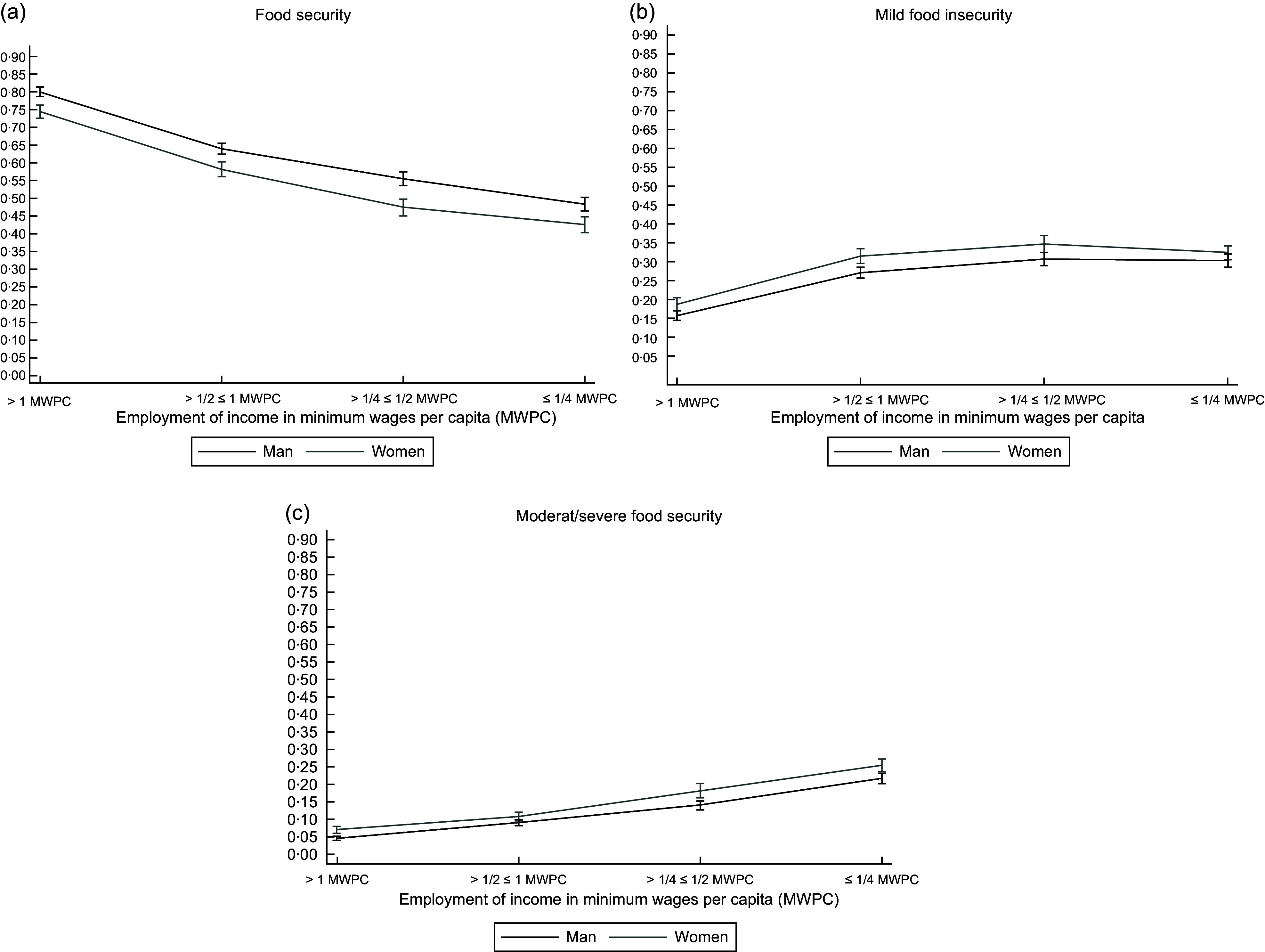
Distribution of predicted probabilities for food security (a) and mild food insecurity (b) and moderate/severe food insecurity (c) according to employment income and the gender of the household reference person. Family Budget Survey – 2018.

When we evaluated the predicted probability of FS (Fig. 2(a)) and levels of FI (Fig. 2(b) and (c)) regardless of work income, the probability of FS was greater in families in which men were the reference person, whereas the probability of moderate/severe FI was significantly greater for families headed by women (Fig. 2).

## Discussion

This study examines the interaction of the gender and employment income of the family’s reference person and FI and reveals a greater probability of moderate/severe FI in families headed by women and those with a lower level of income. This important analysis can explain the implications of gender inequality for food-insecure households in Brazil.

The analysis in this study adopted an intersectional gender perspective^(24–28)^ using the theoretical references of gender and feminist studies. In addition to the binary ‘woman or man’, this study considered the explanatory argument of power relations between the sexes that structure inequalities^(17)^.

Employment income plays a fundamental role in ensuring access to adequate food in terms of quantity and quality, as captured by FS at population measurement scales^(29,30)^. This relationship has been studied by Loopstra and Tarasuk (2013)^(16)^, who analysed the change in the severity of FI in low-income Canadian families. According to these authors, greater employment income gains lead to a reduction in the severity of FI, whereas greater income losses lead to greater limitations due to FI. Recently, Mabli *et al.* (2023)^(31)^ monitored changes in the employment of families in the USA and reported that the experience of reduced income was associated with FI.

In Brazil, despite the extensive literature on family income and FI^(13,14,32)^, we are not aware of previous studies that have directly addressed the relationship between employment income and FI. Bezerra *et al.* (2020)^(33)^ used a social vulnerability index and reported that income, as well as access to and the form of entry into the job market, was associated with FI. The authors proposed a spatial analysis of the distribution of FI in the Brazilian territory with data from the 2004, 2009 and 2014 from the National Household Sample Survey (*Pesquisa Nacional por Amostras de Domicílios* – PNAD) and identified a higher prevalence of FI, higher income and greater work vulnerability in the northeast region and a lower prevalence of FI, lower income and less work vulnerability in the centre-south region of the country^(33)^.

In a previous study that used the same database as this article, the income composition of Brazilian families with severe FI headed by women presented a lower contribution to labour income than that of families headed by men^(34)^. According to the authors, this characteristic occurred in both families with lower incomes and those with higher family incomes in urban and rural territories in all regions of Brazil. Moreover, the contribution of labour income for Brazilian rural families headed by women in conditions of severe FI and severe FI was lower than the contribution for urban families headed by women^(34)^.

In this study, families with a female reference person at all income levels were more vulnerable to moderate/severe FI than families with a male reference person, even at the same income level. Gender inequality in FI has been attributed to inequalities in access to education, employment, productive resources and income, all of which are determinants of FI^(5–8,12,34)^. The allocation of reproductive labour and women’s disproportionate responsibility for tasks essential to social reproduction make women less available for productive labour, especially when this responsibility establishes a class relationship^(17,34)^.

Brazilian women lose almost 2 h a week of paid labour hours because of activities that they perform for free, such as domestic chores and caring for people^(35)^. A continuous workday causes women to work more and dedicate almost twice as much weekly time (21·3) to reproductive labour as men do (11·7)^(35)^. Furthermore, when women are employed, they dedicate approximately seven more hours to domestic work than men do. The inclusion of women in the labour market has little effect on the time men dedicate to reproductive labour, which also contributes to gender inequality in work performed outside the home.

According to the report ‘New Divis Light Data on Gender Disparities in the Labor Market’^(36)^, the overall participation of women (61·4 %) in the labour market remains much lower than that of men (90·6 %), with a difference of 29·2 % in the year 2022^(36)^. Notably, in Brazil, although women represent the majority of the working-age population (51·7 %), the level of female employment is 19·7 % lower than the level of male employment^(35)^.

In addition to time restrictions, reconciling family and paid work contributes to greater mobility restrictions, especially for women in situations of poverty and extreme poverty. Women more often work in informal situations^(36)^. A study of Peruvian families revealed that in female-headed households, more than half of the household members were not formally or independently employed^(37)^. These female-headed households were also three times more likely to have FI than male-headed households were^(37)^. Thus, the sexual division of labour, in addition to compromising the insertion and stability of women in paid work, tends to contribute to women’s greater vulnerability to informal jobs, a lack of social protection, lower income and greater vulnerability to job loss.

In a study conducted in St. Louis County in the USA, black women were more likely to experience job loss and FI during the COVID-19 pandemic^(38)^. Furthermore, black women were more likely to be laid off than white men were and were more concerned about access to food^(38)^. In fact, job loss contributes to economic hardship and can increase families’ risk of FI. Despite the pandemic context, which increases social vulnerability, black women are historically more likely than white women to provide care and support to their families in isolation and while managing multiple responsibilities. Santos *et al.* (2022) analysed the same POF 2018 database and reported that Brazilian families headed by black women experienced moderate/severe FI in greater proportions and that black women were more vulnerable than brown women in all Brazilian regions. Given the scarcity of public policies and adequate social instruments to support black women, it is possible that families headed by black women are more vulnerable to FI.

Considering the conditions of female-headed households highlighted in this study, families in vulnerable conditions may have an advantage in increasing their FS if this improvement depends exclusively on employment. Santos *et al.* (2022)^(37)^ reported that in Peruvian families, 41 % of households headed by women do not have family members who are employed, whereas 23 % of households headed by men do^(34)^. In contrast to the findings of this study that employment income is associated with FI, Santos *et al.* (2022)^(37)^ found that the number of working residents in a family was not associated with FI. A study by McIntyre *et al.* (2012)^(29)^ corroborated the findings of this article and reported that families with working residents have greater FI when the reference person is female.

Importantly, this article presents results from a national survey on the evolution of FS/FI in Brazil before the COVID-19 pandemic. Studies that evaluated the 2018 POF data revealed a significant increase in FI and a decrease in FS in the national territory^(2–4)^, indicating an increase in social inequalities in the country^(14)^ that is associated mainly with gender differences^(5,6,34)^.

The economic crises during the period of analysis used in this study may have limited entry into the job market for both men and women^(39)^. However, in deeply patriarchal societies and institutions, women are more affected by the scarcity of employment opportunities, especially in periods of crisis^(8,38)^. During the health crisis that occurred due to the COVID-19 pandemic, the FI situation for women^(8,37,38)^ was even worse. The pandemic resulted in unemployment, a reduction in the income of informal workers and limited access to social support^(8)^. For women, social isolation measures (which were necessary to contain the virus) also contributed to an increase in unpaid work, domestic tasks and the amount of care needed for patients and children^(38)^.

The POF questionnaire considered seven types of jobs for each resident of a household (i.e. domestic, military, private, public (federal, state or municipal job), employer, self-employment and unemployment job). In this study, the authors did not consider whether the individual income of the reference person in the family was from a formal or informal job. This limits the analysis of gender inequality in labour income. Informal jobs, for example, can contribute to household FI. In this sense, women are more vulnerable to unstable employment.

Another limitation of this study is related to the definition of the reference person for the family. This concept has always been associated with authority and responsibility for the family’s income and, in most cases, the most important source of support. However, over the years, Brazilian family structures have changed, which makes it impossible to define the exact reasons for choosing a reference person^(5)^. In this sense, it is not possible to establish the reason why a woman or a man declares themselves the head of the household. This makes it difficult to establish a relationship between household FI and aspects related to headship, such as living alone, contributing the largest share of income and taking care of the house^(39)^. Nevertheless, the study of the interaction between the sex of the family’s reference person and income from work, which is an important component of family income and, therefore, a determining factor in FI, allows for an unprecedented debate on the inequality of access to income from work in Brazilian families with female household heads and their vulnerability to FI.

A new study that simultaneously evaluates FI according to family income (the next POF) will be released in the coming years. Nevertheless, the results discussed in this study are highly important to the debate on the goals of the Brazil without Hunger Plan (in Portuguese, Plano Brasil Sem Fome), which was implemented in September 2023^(40)^.

### Conclusion

Studies often debate how gender inequalities impact the family income of Brazilian families, thus jeopardising the guarantee of food and nutritional security. In this sense, this study contributes to reiterating that the differences in family income arise from employment income when the woman is the head of the household. As a result, inequality and hunger still persist in Brazil.

This study adopted the approach favoured by gender and feminist studies to identify power relations between women and men that help to structure and reproduce inequalities that threaten the right to food.

The reinforcement of gender inequalities in households through the priority allocation of women’s reproductive labour can be a challenge for the state’s direct actions to combat hunger. In addition to recognising and valuing unpaid work, a government measure can address the socialisation of care, access to work income and the decline of FI in families led by women by guaranteeing accessible public services, such as daycare centres, comprehensive public schools and community/solidarity kitchens. Furthermore, reducing working hours without compromising work performance can provide long-term benefits with respect to the division of domestic and care tasks, thereby preventing overload and women’s vulnerability to FI.

Integrating government policies that not only promote social policies to reduce gender inequalities but also increase labour income under the Brazil Without Hunger Plan will be challenging. However, we believe that doing so will make it possible to achieve the goal of significantly reducing FI in the Brazilian population and ensuring access to healthy, high-quality food for everyone.
